# Histological changes in human skin 32 days after death and the potential forensic significance

**DOI:** 10.1038/s41598-020-76040-2

**Published:** 2020-10-30

**Authors:** Wang Wei, Qi Michu, Dong Wenjuan, Wen Jianrong, Han Zhibing, Yang Ming, Jin Bo, Lin Xia

**Affiliations:** 1 Department of Forensic Medicine, North Sichuan Medical College, Nanchong, 637000 People’s Republic of China; 2 School of Forensic Medicine, Kunming Medical University, Kunming, 650500 People’s Republic of China; 3 Nanchong Public Security Bureau, Nanchong, 637000 People’s Republic of China; 4 Department of Clinical Medicine, North Sichuan Medicine College, Nanchong, 637000 People’s Republic of China; 5 Department of Rehabilitation Medicine, Affiliated Hospital of North Sichuan Medicine College, Nanchong, 637000 People’s Republic of China

**Keywords:** Anatomy, Medical research

## Abstract

To observe the histological changes in human skin within 32 days after death to explore its potential significance in forensic practice. The intact full-thickness skin and subcutaneous tissue from the sternum of eight corpses were placed in an environment of 4–6 °C for 4 h, 6 h, 12 h, 18 h, 24 h, 36 h, 48 h, 60 h, 72 h, 84 h, 96 h, 6 d, 8 d, 10 d, 12 d, 16 d, 20 d, 24 d, 28 d, and 32 d. Then, the whole layer of the skin was stained with haematoxylin & eosin. The histological morphology of the epidermis, dermis and appendages (sweat glands, hair follicles, and sebaceous glands) was observed under an light microscope. The epithelial nucleus condensed at 24 h after death, and cell lysis was exhausted after 20 days. The post-mortem changes in the dermis occurred later than that of the epidermis (72 h), but after epidermal changes started, the change was more rapid. At 16 d, the layers had become homogenized. The epidermis and dermis had completely separated 24 d after death. The changes in the sweat glands appeared earlier (24 h) and disappeared later (32 days); the sebaceous glands and hair follicles began to undergo degenerative changes at 96 h after death, and at approximately 20 d, only their contour remained. There were individual and structural differences in the post-mortem histological changes in the skin. At 4–6 °C ambient temperature, some structures of the human skin still exist for a long time after death, and these structures can be used to identify the source of the tissue; post-mortem histological changes in the skin occur at specific times, which can be used to help infer the time of death. A comprehensive observation of changes in the skin composition/structure is required to comprehensively analyse possible death times.

## Introduction

Accurate estimation of time of death or post-mortem interval (PMI) has always been the focus and a primary difficulty of forensic pathology. People have tried to use many ways to infer the time of death. Changes to the skin occur at different times after the death of the body. The general morphology of the skin after death and the changes in histology, biomechanics^1^, temperature, spectral characteristics^2–4^, microbes^5–9^, skin resistivity^10,11^ and other factors^12,13^ have been studied, and some progress has been made, but it has not been enough to be applied to actual situations and to be used to infer the time of death accurately.

The skin is a relatively long-lived body tissue after death. Previous studies^14,15^ have shown that morphological changes in experimental animals and human skin after death correlate with time. However, until now, there have been few studies^14^ on histological changes in human skin after death, and the time after death involved in most prior studies has been relatively short. This study looked at the histological changes in isolated human skin for a longer time after death to explore their potential significance in forensic practice, such as time of death inference.

## Methods

### Study design and participants

In this study, eight cadavers were randomly obtained, aged between 22 and 33 years old, including two women and six men, BMI 21.2–23.3. One donor died from mechanical asphyxia, and the other seven died from a mechanical injury. The eight donors were healthy before their death, with no skin diseases and no skin damage or scars.

### Materials

Bouin's solution, ethanol, xylene, paraffin (Chengdu Kelong Chemical Reagent Factory, China) and haematoxylin and eosin (Shanghai Fusheng Industrial Co., Ltd., China) were obtained. The slices were viewed using an Olympus BX-43 microscope (Japan). We used cellSens Standard software (Version 1.2.1) to collect the pictures.

### Haematoxylin eosin staining

All cadavers were dissected within four hours of death. The skin in the chest area of the human body was selected in this study as it is a non-articular surface, and the subcutaneous tissue is thinner and less affected by obesity , Sun exposure, friction and damage. The whole layer of the skin and subcutaneous tissue from the sternum angle to the xiphoid process was removed by a surgical blade. The piece was trimmed to 10 cm × 2 cm (length × width), and then, the sample was placed flat in a refrigerator at 4–6 °C. At different PMIs (4 h, 6 h, 12 h, 18 h, 24 h, 36 h, 48 h, 60 h, 72 h, 84 h, 96 h, 6 d, 8 d, 10 d, 12 d, 16 d, 20 d, 24 d, 28 d, and 32 d) a piece measuring 0.3 cm × 1.0 cm was extracted from each sample.

The samples were immersed in Bouin's solution for four days, transferred to 70% ethanol, dehydrated through a serial alcohol gradient, and embedded in paraffin wax blocks. The sample sections were dewaxed in xylene and rehydrated through decreasing concentrations of ethanol. Then, they were subjected to H&E staining. We observed the slices and recorded the results. The changes in the epidermis, dermis, sweat glands, hair follicles and sebaceous glands were observed.

### Ethics

The experiments were carried out in accordance with the Declaration of Helsinki (1983) of the World Medical Association, and the protocols were approved by the Institutional Research Review Board at North Sichuan Medical College (201810634009) with informed consent for study participation from next of kin/ legal representative(LAR).

## Results

The skin samples from the different bodies changed almost uniformly after death. The time and rate of change of the individual samples after death for certain structures were different. The following description is based on the observational results of the eight cadavers.

### Epidermis and dermis (Table 1)

**Table 1:** Histological changes in the human epidermis and dermis 32 days after death.

PMI	Epidermal histology change	Dermis histology change
0–18 h	No obvious change	No obvious change
24 h	The cell layer occasionally showed nuclear pyknosis; no obvious changes were observed in the stratum corneum	No obvious change
36 h	Scattered nuclear fragmentation is seen in the cell layer, showing nuclear pyknosis; the stratum corneum changes as above	No obvious change
48 h	The cell layer occasionally shows nuclear lysis, local visible nuclear pyknosis, rare nuclear fragmentation; local swelling of the stratum corneum	No obvious change
60 h	The cell layer occasionally shows nuclear lysis, local visible nuclear pyknosis, rare nuclear fragmentation; local swelling of the stratum corneum	No obvious change
72 h	The cell layer shows less nuclear pyknosis, visible nuclear fragmentation, rare nuclear dissolution; stratum corneum swelling	There is a small amount of fibre swelling, and there is no obvious change in the whole
84 h	The cell layer shows less nuclear pyknosis, visible nuclear fragmentation, rare nuclear dissolution; stratum corneum swelling	Visible fibre swelling, no significant change in the overall
96 h	Cell layer cell gap widened; more nuclear pyknosis, nuclear fragmentation, nuclear dissolution; stratum corneum changes as above	Visible fibre swelling, no significant change overall
6 d	Cell layer changes as above; the stratum corneum is swollen	Significant swelling of the fibres, visible focal fibre dissolution
8 d	The epidemal layer is thin; the cell layer is basically free of normal cells, with nucleus pyknosis, nuclear fragmentation, and nuclear dissolution; the stratum corneum is swollen, and a purple foam-like stain layer is visible on the surface	The papillary layer fibres are dissolved, the reticulated fibres are arranged in order, the swelling is obvious, and there is focal dissolution
10 d	The local dermis began to separate; the cell layer and stratum corneum changed as above	The papillary layer fibres are dissolved, the reticulated fibres are arranged in order, the swelling is obvious, and there is focal dissolution
12 d	The cells in the cell layer are disorderly, their boundaries are unclear, the number of cells is reduced, and the remaining changes are the same as above	The papillary layer is homogeneous, the woven layer is partially dissolved, and the remaining fibres are swollen
16 d	The number of cells in the cell layer is further reduced, and the remaining changes were the same; there was a contiguous purple-blue staining area at the junction of the epidermis and the dermis; the stratum corneum was severely swollen	Both the papillary layer and the woven layer are homogenized
20 d	The cells in the cell layer are all dissolved, the epidemal is a purple-blue stained area, the stratum corneum changes are the same as above, and the epidemal is mostly separated from the dermis	Both the papillary layer and the woven layer are homogenized
24 d	Completely separated from the dermis, the remaining changes are the same as above	The fibre dissolves into a homogeneous shape, and the papillary layer and the woven layer cannot be distinguished
28 d	Completely separated from the dermis, the remaining changes are the same as above	The fibre dissolves into a homogeneous shape, and the papillary layer and the woven layer cannot be distinguished
32 d	The epidemal disappears, and the purple-blue stained area disappears	Fragmented, flaky

**Figure 1:**
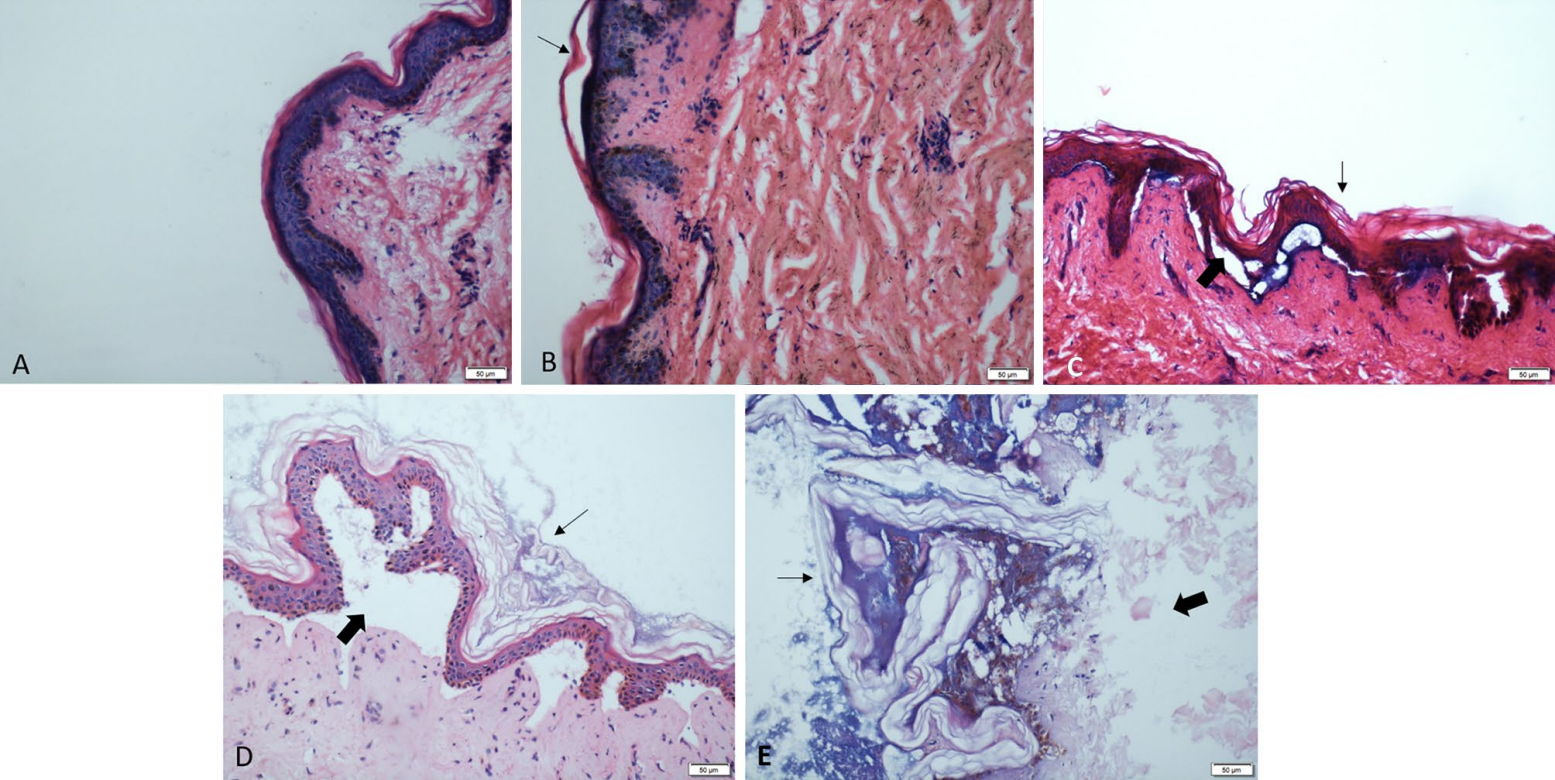
Microphotograph showing the epidermis. (**A**) The epidermal layer shows no obvious change at the time of death. (**B**) Focal nuclear retraction of the epidermis and local swelling of the stratum corneum (small black arrows) 48 h after death. (**C**) Focal surface dermal separation (bold black arrows) 10 days after death. (**D**) Complete dissolution of the epidermal cells, the stratum corneum is severely swollen, and the epidermis is mostly separated from the dermis twenty days after death. (**E**) Complete separation of the epidermis 24 days after death. (H and E, original magnification × 20, small black arrows point to changes in the cuticle, the bold black arrows point to the separation of the dermis and epidermis). (All images are collected by cellSens Standard software whose URL is https://www.olympus-lifescience.com.cn/en/software/cellsens/).

The epidermis is composed of layers of squamous epithelial cells, including the stratum corneum, the transparent layer, the granular layer, the spinous layer, and the basal layer. Because the chest epidermis is thin, the layers are difficult to distinguish completely, and the changes are basically synchronized, so their descriptions are combined. The epidermal layer shows no obvious change at the time of death(Fig. 1A). No obvious changes were observed in the early post-mortem (< 24 h), but nuclear pyknosis began to appear in the cells after 24 h. With the prolongation of PMI, the cell degeneration and gradual changes such as nuclear fragmentation and lysis gradually increased (Fig. 1B). The dermis was focally isolated (Fig. 1C), and on the 20th day, the epidermal cells were all dissolved (Fig. 1D). On the 24^th^ day, the dermis was completely separated (Fig. 1E), and the epidermis disappeared at 32 d.

**Figure 2:**
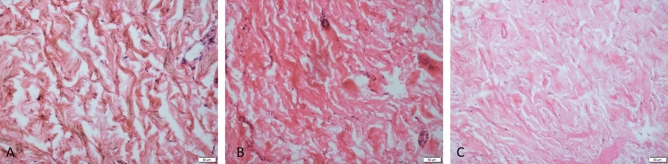
Microphotograph showing the dermis. (**A**) The dermis layer shows no obvious change at the time of death. (**B**) Fibre swelling 96 h after death. (**C**) Homogenization of dermal fibres twenty days after death (H and E, original magnification × 20). (All images are collected by cellSens Standard software whose URL is https://www.olympus-lifescience.com.cn/en/software/cellsens/).

The dermis is mainly composed of connective tissue, which is divided into a papillary layer and a reticular layer that are roughly demarcated by shallow blood vessels. The papillary layer is thin, and it has papillary protrusions (dermal nipples) on the epidermis side, which are intertwined with the epidermis to form a corrugated and firm link. The collagen fibres of the papillary layer are slender and disorderly, and they are only stained lightly with H&E. The dermal mesh is thicker, and the collagen fibre bundles are thick, mostly parallel to the epidermis, and they are intertwined with each other. The H&E staining shows red-dyed corrugations in the longitudinal and transverse directions, which are indistinguishable from the elastic fibres. The early dermal fibre changes were not obvious shortly after death (Fig. 2A). At 72 h, some fibre swelling was observed, and it gradually increased (Fig. 2B). Focal fibre dissolution was observed by the sixth day, and by the 16 d, dermal fibres were observed, and extensive homogenization had occurred (Fig. 2C).

### Sebaceous glands, sweat glands and hair follicles (Table 2)

**Table 2:** Histological changes in sweat glands, sebaceous glands and hair follicles with time of death.

PMI	Sweat glan	Sebaceous gland	Hair follicle
0–24 h	24-h vacuoles began to appear in the cytoplasm	No obvious change	No obvious change
36 h	Interstitial swelling occurs; the sweat gland epithelial cells become smaller	No obvious change	No obvious change
48 h	Interstitial swelling occurs; the sweat gland epithelial cells become smaller	No obvious change	No obvious change
60 h	The gland tube becomes smaller, the interstitial is swollen, and the focal cytoplasm has vacuoles	No obvious change	No obvious change
72 h	The nucleus is swollen and the cytoplasm has vacuoles	No obvious change	No obvious change
84 h	The cavity in the centre of some sweat glands disappears, the basement membrane of the sweat gland is locally swollen, and the epithelial cells are focally separated from the basement membrane	No obvious change	Occasionally, under high magnification, the cells are swollen, the hair follicles are dissolved at the roots, and the inner and outer layers of the hair follicles are separated (longitudinal section)
96 h	Epithelial cell disorder, a small amount of nuclear pyknosis, nuclear fragmentation, nuclear lysis; cell and basement membrane detachment	The sebaceous cell nucleus is irregular, and the cytoplasm begins to show flocculation changes	Hair follicle cells visible, nuclear pyknosis, nuclear fragmentation, nuclear dissolution
6 d	Epithelial cells have more nuclear pyknosis, nuclear fragmentation, nuclear lysis, and unclear cell boundaries	The sebaceous cell nucleus is dissolved, and the cytoplasm is flocculated	The gap of the hair follicle cells is widened, and the remaining changes are the same as above
8 d	Epithelial cells have more nuclear pyknosis, nuclear fragmentation, nuclear lysis, and unclear cell boundaries	The sebaceous cell nucleus is dissolved, and the cytoplasm is flocculated	The gap of the hair follicle cells is widened, and the remaining changes are the same as above
10 d	The cell hierarchy is severely disorganized, the structure of the sweat glands can still be distinguished, no normal epithelial cells, solid shrinking of the nuclei, nuclei are broken up, karyolysis	Sebaceous cell nucleus disappears	The number of hair follicle cells is reduced, and the remaining changes are the same as above
12 d	The cell hierarchy is severely disorganized, the structure of sweat glands can still be distinguished, no normal epithelial cells, shrunken solid nuclei, nuclei are broken up, karyolysis	Sebaceous cell nucleus disappears	The number of hair follicle cells are further reduced, showing more nuclear pyknosis, nuclear fragmentation, and nuclear dissolution
16 d	Residual sweat gland structure	Most of the sebocytes and basal cells are dissolved, leaving a reddish floc	Most of the hair follicle cells dissolve and disappear, leaving a small amount of nuclear pyknosis, nuclear fragmentation
20 d	Residual sweat gland structure	Most of the sebocytes and basal cells are dissolved, leaving a reddish floc	Only a few cells present, blurred hair follicle outline
24 d	Residual sweat gland structure	Sebocytes and basal cells are completely dissolved, only the sebaceous gland outline	No hair follicle structure
28 d	Residual sweat gland structure	No sebaceous gland structure	No hair follicle structure
32 d	No sweat gland structure	No sebaceous gland structure	No hair follicle structure

**Figure 3:**
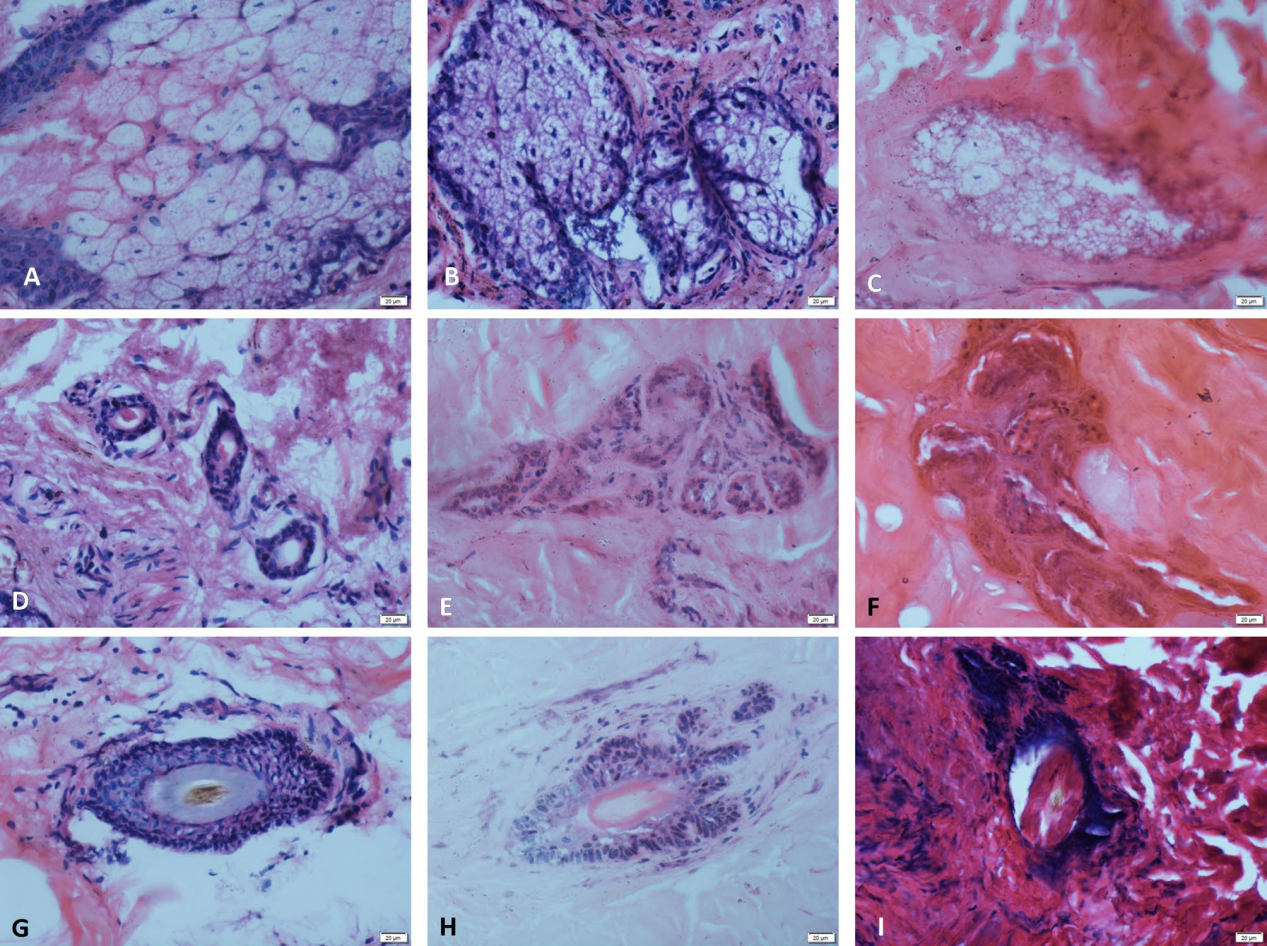
Microphotograph showing the sweat glands, sebaceous glands and hair follicles. (**A**, **D**, **G**) The sebaceous glands, sweat glands and hair follicles show no obvious change at the time of death. (**B**) Cytoplasmic changes in flocculation eight days after death. (**C**) Sebaceous cell nucleus disappears 10 days after death. (**E**) After 12 days of death, sweat glands lose most of their normal cells. (**F)** Residual sweat gland structure twenty days after death. (**H**) Cell gap widening six days after death. (**I**) Only the outline of the hair follicle can be seen 20 days after death (H and E, original magnification × 40). (All images are collected by cellSens Standard software whose URL is https://www.olympus-lifescience.com.cn/en/software/cellsens/).

The sebaceous glands are closely related to the hair follicles. Most of the sebaceous glands open in the funnel of the hair follicle. The peripheral part of the sebaceous gland is the basal cell, and the inside consists of sebocytes.The sebaceous glands show no obvious change at the time of death (Fig. 3A). Before 84 h, no significant structural changes were seen in the sebaceous glands. From 96 h, the nucleus of the sebocytes gradually dissolved and disappeared, and the cytoplasm changed and increased (Fig. 3B,C). At 24 d, the sebocytes and basal cells were completely dissolved. At 28 d, no sebaceous gland tissue was seen.

The sweat gland is divided into a secretory part and a duct. The secretory part is composed of a single layer of cubic epithelium. The single layer of cubic epithelium is composed of dark cells and clear cells, which are surrounded by myoepithelial cells. The ductal epithelium is composed of two layers of small, basophilic cuboidal cells, while the lumen is lined by a ring of uniform eosinophilic utricles. The sweat gland show no obvious change at the time of death (Fig. 3D). After 24 h, vacuoles appeared in the cytoplasm of the sweat gland cells. After that, the degeneration of the cells increased, and it was most obvious at 10–12 days (Fig. 3E). On the 16th day, only the residual sweat gland structure was observed (Fig. 3F), and no sweat gland structure was observed on the 32nd day.

The hair follicle is divided into three parts: the opening of the sebaceous gland duct and the attachment of the pilose muscle: the funnel, the isthmus and the lower part of the hair follicle. The epithelium of the funnel of the hair follicle is connected with the epidermis, and the morphology is exactly the same. The lower part of the hair follicle is swollen into a ball, and the centre has an inward depression, that is, a dermal papilla. The hair follicle show no obvious change at the time of death (Fig. 3G). No significant change was observed before 72 h. At 16 d, most of the hair follicle cells had dissolved and disappeared. No hair follicle structure was seen from 24 d (Fig. 3H,I).

## Discussion

The theory and technique of histology and pathology is the basis of classical forensic science. H&E staining is a simple, reliable and economical technical means to observe histomorphology^15^. Kovarik^16^, studied the appearance of three corpses in the outdoors, dark, at 3–25 °C in the early post-mortem (one week) period by skin microscopy and found that epidermis and dermis separation occurred four to six days after death. The degeneration of the sweat glands was obvious at five days after death, and the dermis degeneration appeared two days after death. Bardale et al.^14^ studied the histological changes in post-mortem skin of 30 human cadavers at 23–37 °C. Their study showed that after nine to 12 h of death, the basal cells of the epidermis began to deform, and the surface and dermis were partially separated. After 12–18 h of death, the epidermis and dermis were separated, and the dermis began to separate 18 h after death. The sweat gland cells underwent vacuolar degeneration four hours after death, with an increase of PMI, and the amount of cell vacuolar degeneration increased. At approximately 15 h, the cells ruptured; after 18 h of death, the sebaceous glands increased with PMI, the sebocytes lost their cell structure and the nuclei dissolved; the hair follicles died 18 h after death. There were degenerative changes, and the papilla was broken down.

The post-mortem changes in the skin in the chest area of our eight corpses were generally consistent with those observed by Kovarik and Bardale, but the time of appearance was relatively late, and the rate of change was slower. A long time after death, they were still observed. Some structures of the skin, hair follicles, sebaceous glands, and sweat glands still partially survived after 20–28 days of death, which could be related to the lower ambient temperature (4–6 °C) of the skin samples in our study. Thus, at certain ambient temperatures, certain structures of human skin are still identifiable for a long time after death, which has potential applications in the identification of the sources of biological tissue fragments.

The observations in our study suggest that post-mortem changes in the epidermis, dermis, and skin appendages show a certain degree of time compliance, such as the changes in the epidermal cells and hair follicle cells. The changes in these two within 84 h after death were not obvious. Then, changes such as nuclear pyknosis karyorrhexis and karyolysis occurred as the PMI increased. On the 20th day after death, the cells of these two structures were almost completely dissolved. The hair follicles continue from the epidermis, especially the cell layer of the follicular infundibulum, which is the same as the epidermis, which explains the synchrony of the changes.

At some specific time points, the structure of the skin has prominent characteristics, such as at 16 days after death. At this time, the stratum corneum of the skin epidermis is obviously swollen, and the cells have obvious nuclear pyknosis and fragmentation. There is a junction between the epidermis and the dermis. In the contiguous purple-blue staining area, the dermal fibres are homogenized, the cells of the sebaceous glands and hair follicles are mostly dissolved, and only the remaining sweat gland structure is seen. Observation of the above histological changes depends on the experience and concentration of the examiner. The description of the changes under the microscope is difficult to quantify, so there may be some observer bias.

The variation in the human skin with the time of death can be used as an auxiliary means for inferring PMI. Our study, it was also found that the individual samples had different appearance times and rates of change after death, that is, individual differences and structural specificity after death. Therefore, it is speculated that PMI can not only be based on individual specific skin structure changes but should also be comprehensively observed. The composition and structure of the skin can be subjected to a comprehensive analysis.

In practice, the body has its own individual characteristics, such as sex, age, height, weight, disease, and cause of death. After death, it will be in various environmental conditions, such as temperature, humidity, air flow, water flow, insects, animal activities, etc. These factors will affect the post-mortem changes in the body. Our study investigated the histological changes in excised skin of eight cadaveric bodies at a lower ambient temperature and a longer time after death, filling in some gaps not covered by previous studies, but there are still many problems worth exploring, such as observing different individual characteristics, changes in the body under different environmental conditions after death, special staining to highlight a specific tissue structure, and quantitative analysis of specific microscopic targets to establish a quantifiable association between histological changes and time after death, all of which will be conducive for a more accurate inference of PMI.

In this study, there were only eight cadavers, which is a small number. In a future study, we will increase the sample size. In addition, the age range of the samples selected for this study was 22 and 33 years old, which is relatively limited, so the age range should be further broadened in future studies.
